# The COVID-19 Pandemic and Daily Steps in the General Population: Meta-analysis of Observational Studies

**DOI:** 10.2196/40650

**Published:** 2023-05-30

**Authors:** Ziying Wu, Yilun Wang, Yuqing Zhang, Kim L Bennell, Daniel K White, Liusong Shen, Wei Ren, Jie Wei, Chao Zeng, Guanghua Lei

**Affiliations:** 1 Department of Orthopaedics Xiangya Hospital Central South University Changsha China; 2 Division of Rheumatology, Allergy, and Immunology, Department of Medicine Massachusetts General Hospital, Harvard Medical School Boston, MA United States; 3 The Mongan Institute Massachusetts General Hospital Harvard Medical School Boston, MA United States; 4 Centre for Health, Exercise and Sports Medicine The University of Melbourne Melbourne Australia; 5 Department of Physical Therapy University of Delaware Newark, DE United States; 6 Health Management Center Xiangya Hospital Central South University Changsha China; 7 Hunan Key Laboratory of Joint Degeneration and Injury Xiangya Hospital Central South University Changsha China; 8 National Clinical Research Center of Geriatric Disorders Xiangya Hospital Central South University Changsha China

**Keywords:** COVID-19, daily steps, physical activity, meta-analysis

## Abstract

**Background:**

The COVID-19 pandemic has the potential to accelerate another pandemic: physical inactivity. Daily steps, a proxy of physical activity, are closely related to health. Recent studies indicate that over 7000 steps per day is the critical physical activity standard for minimizing the risk of all-cause mortality. Moreover, the risk of cardiovascular events has been found to increase by 8% for every 2000 steps per day decrement.

**Objective:**

To quantify the impact of the COVID-19 pandemic on daily steps in the general adult population.

**Methods:**

This study follows the guidelines of the MOOSE (Meta-analysis Of Observational Studies in Epidemiology) checklist. PubMed, EMBASE, and Web of Science were searched from inception to February 11, 2023. Eligible studies were observational studies reporting monitor-assessed daily steps before and during the confinement period of the COVID-19 pandemic in the general adult population. Two reviewers performed study selection and data extraction independently. The modified Newcastle-Ottawa Scale was used to assess the study quality. A random effects meta-analysis was conducted. The primary outcome of interest was the number of daily steps before (ie, January 2019 to February 2020) and during (ie, after January 2020) the confinement period of COVID-19. Publication bias was assessed with a funnel plot and further evaluated with the Egger test. Sensitivity analyses were performed by excluding studies with low methodological quality or small sample sizes to test the robustness of the findings. Other outcomes included subgroup analyses by geographic location and gender.

**Results:**

A total of 20 studies (19,253 participants) were included. The proportion of studies with subjects with optimal daily steps (ie, ≥7000 steps/day) declined from 70% before the pandemic to 25% during the confinement period. The change in daily steps between the 2 periods ranged from –5771 to –683 across studies, and the pooled mean difference was –2012 (95% CI –2805 to –1218). The asymmetry in the funnel plot and Egger test results did not indicate any significant publication bias. Results remained stable in sensitivity analyses, suggesting that the observed differences were robust. Subgroup analyses revealed that the decline in daily steps clearly varied by region worldwide but that there was no apparent difference between men and women.

**Conclusions:**

Our findings indicate that daily steps declined substantially during the confinement period of the COVID-19 pandemic. The pandemic further exacerbated the ever-increasing prevalence of low levels of physical activity, emphasizing the necessity of adopting appropriate measures to reverse this trend. Further research is required to monitor the consequence of long-term physical inactivity.

**Trial Registration:**

PROSPERO CRD42021291684; https://www.crd.york.ac.uk/prospero/display_record.php?RecordID=291684

## Introduction

Physical activity has been crucial for preventing and treating multiple chronic health conditions [1,2] and premature mortality [3,4], while the number of daily steps is a primary proxy measure for physical activity [5]. Daily step goals can be tailored to meet individual needs and when used with a monitor are effective in promoting physical activity [6]. Previous studies have found that higher daily steps are associated with multiple health benefits, such as lower risk of cardiovascular disease [7], diabetes [8,9], and all-cause mortality [10-13]. For example, the risk of all-cause mortality in adults shows a declining trend with an increase in daily steps and plateaus at approximately 7000 steps per day [12,13]. In contrast, a decrease in daily steps is responsible for a range of unhealthy outcomes, such as increased risk of cardiovascular disease [14], compromised muscle metabolism [15], and elevated systemic inflammation [15,16]. Specifically, the risk of cardiovascular events was reported to increase by 8% for every 2000 steps per day decrement [14]. A sharp reduction in daily steps might also reduce leg lean mass and induce impairments of myofibrillar protein synthesis in healthy elderly individuals, accompanied by increased circulating inflammatory markers [15,16].

Although global responses were far from homogeneous, most countries adopted restriction measures in varying forms to limit the transmission of COVID-19, such as physical distancing, working from home, and closing schools [17]. These measures have resulted in unprecedented changes in all aspects of daily life [18], and physical activity is certainly one of the aspects being most affected. Recently, several studies reported a significant decline in daily steps during the confinement period of the COVID-19 pandemic [19-23]. One study found that the daily steps of young adults in Singapore decreased by 42% on average during the lockdown period compared with the time before the pandemic [20]. Another study reported a sharp, 30% reduction in daily steps in Chinese citizens and a significant increase in the proportion of adults with frequent low daily steps (≤1500 steps/day for ≥14 days) during the confinement period [19]. By contrast, one study reported only a 5% reduction in daily steps 30 days after the start of lockdown in Australia [22]. These findings indicate the possibility of geographically specific variations in the relationship between COVID-19–related restrictions and daily steps, which limits the generalization of conclusions drawn from single country–based analyses. Therefore, a systematic review is needed to consolidate the current understanding of the impact of COVID-19 on daily steps in order to enlighten future interventions and research directions. In addition, a few studies have also evaluated whether the impact of COVID-19 on physical activity varied by gender. One study suggested that men had more reduction in time spent in moderate-vigorous physical activities and a larger increase in sedentary behaviors than women during the pandemic [24]. However, another study failed to confirm this finding [25].

To fill this knowledge gap, we conducted a meta-analysis of observational studies to quantify both global and regional impacts of confinement during the COVID-19 pandemic on daily steps in the general adult population, and we also examined whether the decline in daily steps differed between men and women.

## Methods

### Protocol and Registration

The MOOSE (Meta-analysis Of Observational Studies in Epidemiology) [26] and PRISMA (Preferred Reporting Items for Systematic Reviews and Meta-Analyses) 2020 [27] guidelines were followed for reporting this study (Multimedia Appendix 1). The protocol for this study was prospectively registered in the PROSPERO database (CRD42021291684).

### Search Strategy

A preliminary search was conducted on PubMed, EMBASE, and Web of Science to retrieve studies that reported daily step data both before and during the confinement period of the COVID-19 pandemic in the general adult population published before November 22, 2021. Further, an updated search was performed on February 11, 2023, to add recently published articles. The detailed search strategy is listed in Multimedia Appendix 2 [28-31]. The International Standard Randomized Controlled Trial Number Register (ISRCTN) and the World Health Organization COVID-19 database were also searched for potential gray literature. The reference lists and citing articles of relevant literature on this topic were also checked to find possible studies that could be included in this research. The language of publication was not restricted.

### Eligibility Criteria and Study Selection

We included observational studies that reported daily steps before and during the confinement period of the COVID-19 pandemic in the general adult population. The reference period was restricted to ≤2 years before the time points when the government implemented the confinement measures. Further eligibility criteria are listed in Textbox 1.

**Eligibility criteria for study screening.**

**Inclusion criteria**

Study type: Observational studies

Participants: General adult population (average age over 20 years)

Outcomes: Daily steps, measured using monitoring devices including wearable physical activity trackers (eg, pedometers, accelerometers, smartwatches, and smart bands) and smartphones mobile apps (eg, iPhone Health, Google Fit, WeChat, and Exercise Health)

Other criteria: Daily step data collected both before and during the confinement period of COVID-19. The reference period was restricted to ≤2 years before the time points when the government implemented the confinement measures

**Exclusion criteria**

Study type: Any non–observational study (eg, reviews, randomized controlled trials, and case reports), conference abstracts

Participants: Children, adolescents, athletes, specific patient groups, participants with particular conditions (eg, pregnant women or elderly individuals at high risk of falls)

Outcomes: Data other than daily steps (eg, physical activity measured using questionnaires alone or data measured by wearable devices that are classified as light, moderate, or vigorous physical activity)

Other criteria: Studies offering financial incentives that may promote physical activity (eg, rewards for individuals who achieve predetermined goals through monitors), studies lacking available data for meta-analysis

Two independent reviewers (LS and WR) screened all titles and abstracts. The full text of potentially relevant literature was further evaluated. A third reviewer (ZW) arbitrated the decision if a consensus was not reached.

### Data Extraction

Two authors (LS and WR) extracted the following data independently: study design, country and continent (Africa, Asia, Europe, North America, South America, and Oceania) where the study was conducted, the sample size used for daily step analysis, participant characteristics (ie, mean age and female ratio), monitoring devices used to measure daily steps, the algorithm used to analyze daily steps, data collection duration, and COVID-19 restriction measures by the government at the time of data collection. The outcomes of interest were the total number of daily steps before and during the confinement period of COVID-19 and the percentage change between the 2 periods. If not reported, the mean (SD) of the outcome was estimated based on the methods provided by the Cochrane Handbook [32]. If data needed to be extracted from graphs, the GetData digitizer was used [33]. This tool has been validated and has good accuracy and precision for data extraction; it has been used in meta-analysis by previous studies [34]. Disagreements, if any, were resolved by consulting with a third reviewer (ZW). When any desired information related to daily steps was missing or unclear, the corresponding author was contacted via email to request or confirm the relevant data, as appropriate. If the data could not be obtained, the study was excluded from the meta-analysis.

### Risk of Bias and Quality of Individual Studies

The modified Newcastle-Ottawa Scale (NOS) for applicable observational studies was used to assess the methodological quality and risk of bias in each study [28-30]. Specifically, the modified NOS consists of 3 domains: *selection* (maximum 3 stars), *comparability* (maximum 2 stars), and *outcome* (maximum 2 stars). Therefore, 1 study can be awarded a maximum of 7 stars. Studies with 6 to 7 stars were rated as high quality, those with 5 stars were rated as moderate quality, and those with 4 or fewer stars were rated as low quality [29]. Two reviewers (LS and ZW) rated the methodological quality of each study independently, and discrepancies, if any, were discussed for consensus or resolved by consulting a third reviewer (YW).

### Data Synthesis and Analysis

A random effects meta-analysis was conducted using Stata software (version 14.0; StataCorp). The change in daily steps was calculated using the mean difference (MD) with the 95% CI between the mean (SD) of daily steps before and during the confinement period of COVID-19. The Cochran Q statistic of the inconsistency index (*I*^2^) was used to test statistical heterogeneity across studies [32,35]. An *I*^2^ value of more than 75% indicated high heterogeneity [32]. Publication bias was assessed with a funnel plot and further evaluated with the Egger test [36]. To test the robustness of the findings on the primary outcome (ie, the number of daily steps), we performed 3 sensitivity analyses. First, we conducted an analysis that eliminated each study one at a time. Second, we performed an analysis that removed the studies rated as having low methodological quality. Third, we performed an analysis that excluded the studies with a small sample size (less than 140) based on the recommendations of Dunton and colleagues [37]. Finally, we also conducted subgroup analyses according to country, continent, and gender.

## Results

### Study Selection

**Figure 1:**
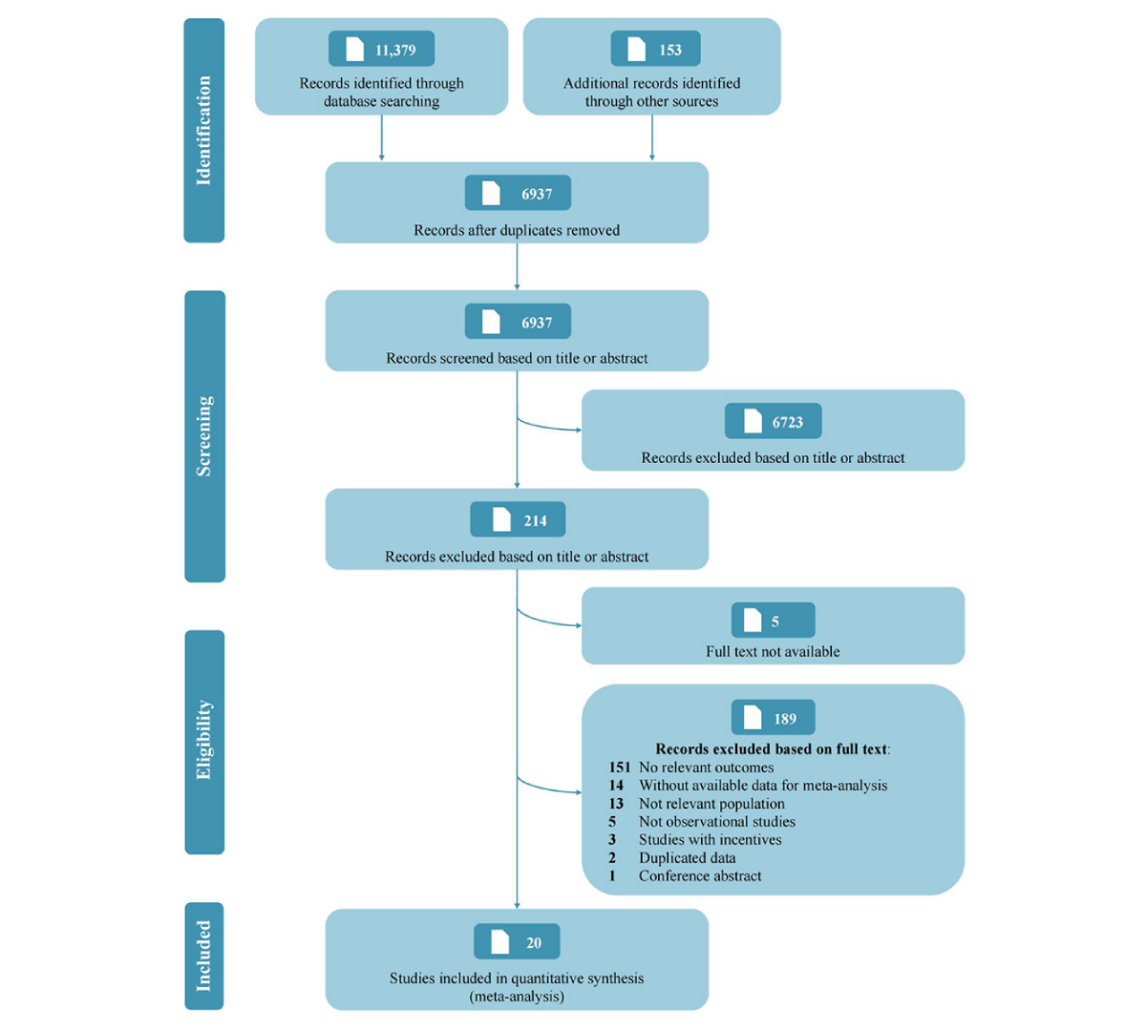
PRISMA (Preferred Reporting Items for Systematic Reviews and Meta-Analyses) flow diagram.

A total of 6937 unduplicated records were preliminarily retrieved, and 214 potentially eligible studies were identified after the title and abstract screening. Then, 5 studies were excluded because the full text was unavailable, and full text assessments were made of the remaining 209 articles. Another 189 articles were eliminated. Of these, 151 studies did not report relevant outcomes (ie, daily steps), 14 did not provide necessary data for meta-analysis, 13 focused on irrelevant populations, 5 were not observational studies, 3 were conducted with financial incentives, 2 reported duplicated data, and 1 was a conference abstract. The remaining 20 studies were included in the meta-analysis [19,20,37-54]. Figure 1 shows the PRISMA flow diagram.

### Study Characteristics

The included studies were conducted in Australia [38], Canada [39], China [19,40-43], France [38], Italy [44], Japan [45-48], Norway [49], Singapore [20], South Korea [50], Spain [51], the United Kingdom [38,52], and the United States [37,38,53,54], and all of them investigated the change in daily steps in the early stage of COVID-19 (from January to September 2020). The reference pre–COVID-19 period was from January 2019 to March 2020. The detailed duration of observation for each study is shown in Table 1. Nine studies measured daily steps with smartphones [19,37,40,41,43,45,46,50,54], 8 with wearable activity trackers [20,38,39,47,48,51-53], and 2 with both wearable activity trackers and smartphones [44,49]; 1 study did not specify the measurement device used in the survey [42]. One study provided 4 groups of data from 4 countries separately [38]. Therefore, the 20 studies included a total of 19,253 participants, and 23 sets of data were included in the meta-analysis. Additional study characteristics are summarized in Table 1.

**Table 1:** Characteristics of studies and participants included in the meta-analysis.

Study	Country/Continent	Participants, n	Age (years)	Female, %	Device	Algorithm	Definition of restriction measures	Observation period	Reference period
Azuma et al [45] 2021	Japan/Asia	530^a^	—^b^	75.1	Smartphone (iPhone Health)	Averaged data from 9 weekly averages	Declared emergency (closed schools and “stay at home” recommendation issued)	Feb 24 to Apr 26, 2020	Feb 24 to Apr 26, 2019
Bird et al^c^ [52] 2021	UK/Europe	190	Range 18-85	77.9	Mobile device (eg, smartwatch)	—	Lockdown	March to May 2020	Before March 2020
Buoite Stella et al [44] 2021	Italy/Europe	197	—	—	Smart technology devices (eg, smartphone, smart band, and smartwatch)	Weekly average	Lockdown	Mar 23 to Mar 29, 2020	One week in Jan 2020
Ding et al^c^ [40] 2021	China/Asia	301	—	76.4	Smartphone (WeChat)	Averaged data from 14-day data	Lockdown	Mar 11 to Mar 24, 2020	Dec 28, 2019, to Jan 10, 2020
Dunton et al [37] 2020	US/North America	149	Range 18-74	—	Smartphone (iPhone Health)	Monthly average	Declared “shelter in place” and “stay at home” orders	Apr 2020	Feb 2020
Gollwitzer et al [54] 2022	US/North America	220	Mean 35.2	41.4	Smartphone (iPhone Health)	Weekly average	Social distancing	Apr 3 to Apr 9, 2020	Apr 3 to Apr 9, 2019
He et al [41] 2020	China/Asia	339	Women: mean 37.6; men: mean 36.4	53.4	Smartphone (eg, Exercise Health, Keep)	Average data during the observation period	Semilockdown (assembly was suspended, and curfew and quarantine measures were implemented in some districts)	Jan 27 to Mar 1, 2020	Dec 23, 2019, to Jan 26, 2020
Henriksen et al^d^ [49] 2021	Norway/Europe	113	Mean 40.6	56.2	Wearable activity trackers (Apple, Fitbit, Garmin, Oura, Polar, Samsung, and Withings), and smartphones (Google Fit and Apple Health)	Monthly average	Lockdown	Mar 13 to Mar 31, 2020	Mar 2019
Hudgins et al [53] 2021	US/North America	80	Mean 32.2	—	Wearable activity trackers	Monthly average	Academic break in 2020, during which the university transitioned to remote learning	30 days after spring break 2020	30 days before spring break 2020
Ji et al [42] 2022	China/Asia	781	—	—	—	—	—	—	—
Karageorghis et al [38] 2021	US/North America	1029	Mean 40.7	76.4	Electronic devices	Self-reported daily steps based on devices’ records	Declared “stay at home” order	Mar 21 to Apr 7, 2020	Before Mar 21, 2020
Karageorghis et al [38] 2021	UK/Europe	392	Mean 51.2	80.1			National lockdown	After Mar 23, 2020	Before Mar 23, 2020
Karageorghis et al [38] 2021	France/Europe	734	Mean 37.7	76.0			National lockdown	After Mar 16 2020	Before Mar 16 2020
Karageorghis et al [38] 2021	Australia/Oceania	386	Mean 42.5	72.5			Interstate border closures^e^	After Mar 19, 2020	Before Mar 19, 2020
Obuchi et al [46] 2021	Japan/Asia	2587	—	76.7	Smartphone	Weekly average	Declared emergency (closed schools and issued “stay at home” recommendation)	Apr 21 to Apr 27, 2020	Apr 30 to May 6, 2019
Ong et al^c^ [20] 2021	Singapore/Asia	1824	Mean 30.9	51.6	Wearable activity tracker (Fitbit)	Averaged data from 3-week data	Lockdown	Apr 7 to Apr 27, 2020	Jan 2 to Jan 22, 2020
Park et al [50] 2021	South Korea/Asia	1163^a^	Mean 23.7	45.6	Smartphone (iPhone Health)	Monthly average	Level 2.5 (gatherings of more than 50 people indoors and more than 100 people outdoors were prohibited)	Sep 2020	Jan to Dec 2019
Sañudo et al [51] 2020	Spain/Europe	20	Mean 22.6	47	Xiaomi Mi Band 2 wrist-worn accelerometer	Weekly average	Lockdown	One week, between Mar 24 and Apr 3, 2020	One week in Feb 2020
Sato et al [47] 2022	Japan/Asia	2846	Mean 45.9	59.6	Accelerometer (Panasonic)	Average data during the observation period	Declared emergency (closed schools and issued “stay at home” recommendation)	Apr 7 to May 13, 2020	Jan 1 to Feb 29, 2019
Wang et al^c^ [19] 2020	China/Asia	3544	Mean 51.6	34.6	Smartphone (WeChat)	Monthly average	Declared emergency (physical distancing measures were issued)	Jan 22 to Feb 20, 2020	Feb 2 to Mar 3, 2019
Woodruff et al [39] 2021	Canada/North America	121	Mean 36.2	80	Wearable activity trackers (Apple, Fitbit, Samsung, Garmin)	—	Declared emergency (physical distancing measures were issued)	Based on the date each person began physically distancing/self-isolating	Based on the date each person began physically distancing/self-isolating
Yamada et al [48] 2023	Japan/Asia	678	—	—	Wearable activity tracker (EW-NK63)	Monthly average	Declared emergency (closed schools and issued “stay at home” recommendation)	April 2020	April 2019
Zhu et al [43] 2021	China/Asia	1029	Range 18-76	69.1	Smartphone (WeChat)	Self-reported daily steps based on smartphone’s records	Lockdown	Three months after entering isolated life	Jan 21 to Jan 25, 2020

^a^Observations are reported instead of participants for these studies, because of uncertainty about the number of participants who were double counted (before and after the confinement period).

^b^Not available.

^c^Data for daily steps were obtained from the authors for these studies.

^d^Only 106 of 113 participants provided characteristics in this study.

^e^“Interstate border closures” means that Australia closed its borders to all noncitizens and nonresidents. This started on March 20, 2020, with no exceptions for Australian citizens, permanent residents, or their immediate families. Furthermore, a lockdown policy started in Australia on March 23, 2020.

### Quality Assessment

Thirteen studies (65%) were rated as having moderate to high methodological quality (Multimedia Appendix 2, Table S1). Considering the widespread weaknesses of small sample size, imbalanced gender ratio, limited age range of the study sample, and poor nationwide representativeness, only 2 studies were rated as having national representativeness of the exposed cohort (ie, samples during the confinement period of COVID-19). Thus, 10% of the included studies received 3 stars in the *selection* dimension, 30% received 2 stars in the *comparability* dimension, and 60% received 2 stars in the *outcome* dimension.

### Quantitative Synthesis

**Figure 2:**
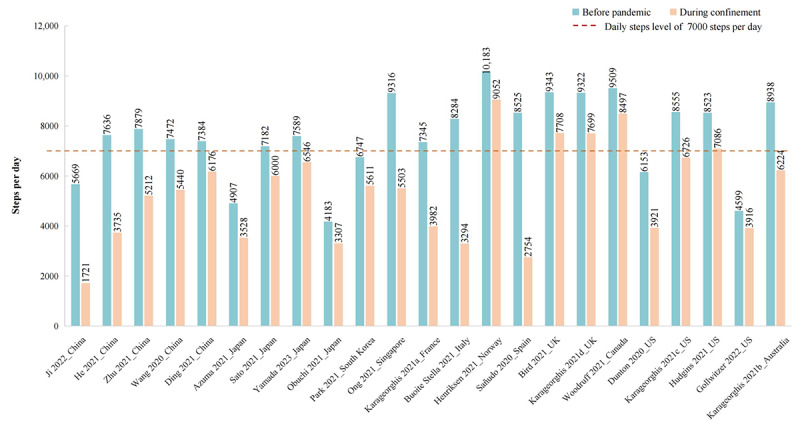
Average daily steps around the period before and during the confinement period across studies.

**Figure 3:**
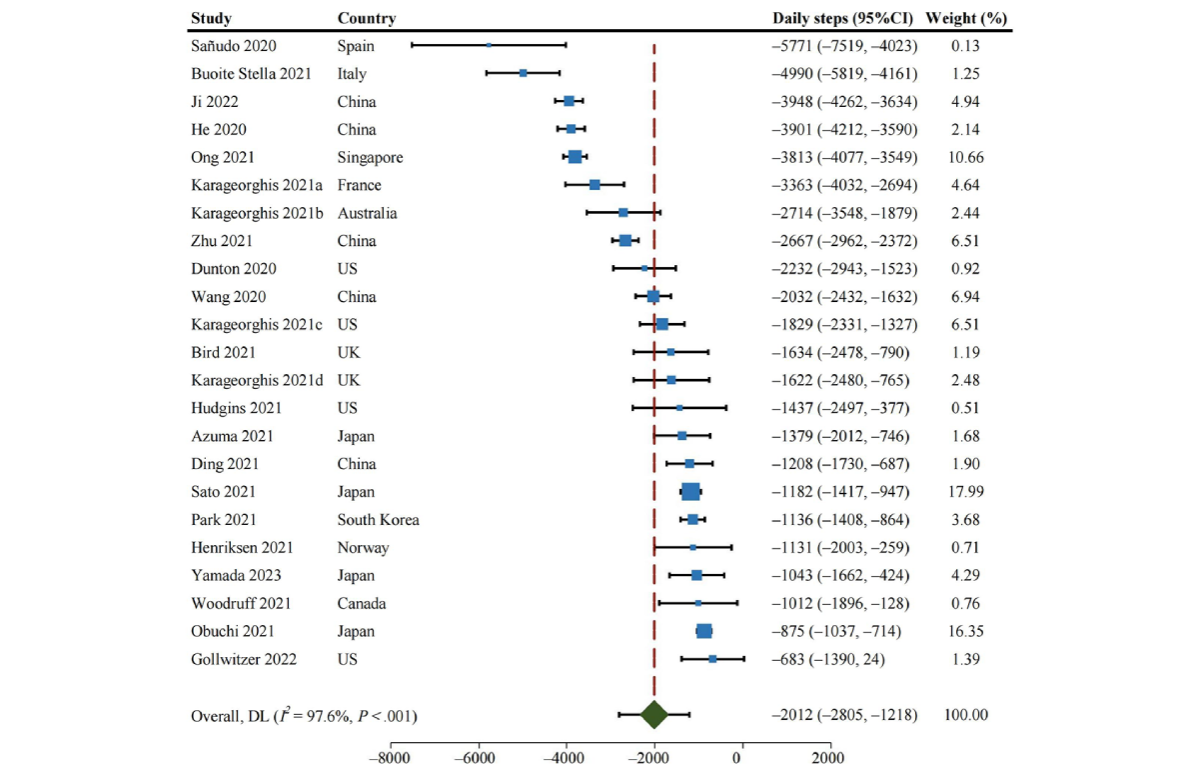
Forest plot of reduction in daily steps during the confinement period. DL: DerSimonian and Laird method.

**Figure 4:**
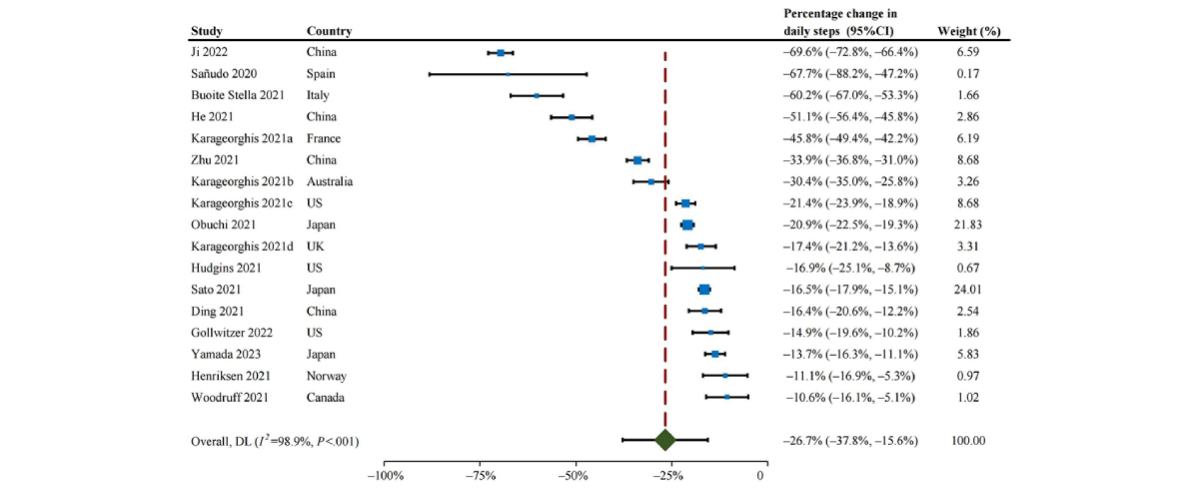
Percentage reduction in daily steps during the confinement period of the COVID-19 pandemic. DL: DerSimonian and Laird method.

As shown in Figure 2, the proportion of studies with subjects with optimal daily steps (ie, ≥7000 steps per day) declined from 70% before the pandemic to 25% during the confinement period. The change in daily steps between the 2 periods ranged from –5771 to –683. The pooled MD from the random effects pooled analysis was –2012 (95% CI –2805 to –1218; Figure 3), with significant heterogeneity among studies (*P*<.001; *I*^2^=97.6%). The funnel plot shows a symmetrical horizontal distribution for each study relative to the vertical line, suggesting the absence of reporting bias (Multimedia Appendix 2, Figure S1). The Egger publication bias indicated a lack of publication bias (Multimedia Appendix 2, Figure S2). Sensitivity analysis, performed by omitting each study one by one, revealed that the change in daily steps was not significantly altered by any individual study (Multimedia Appendix 2, Figure S3). Moreover, the results remained robust in sensitivity analyses after removing low-quality studies (MD=–1714, 95% CI –2809 to –618) and after removing small-sample studies (MD=–2024, 95% CI –2836 to –1212). The percentage change in daily steps varied from –69.6% to –10.6% across different studies (Figure 4).

**Figure 5:**
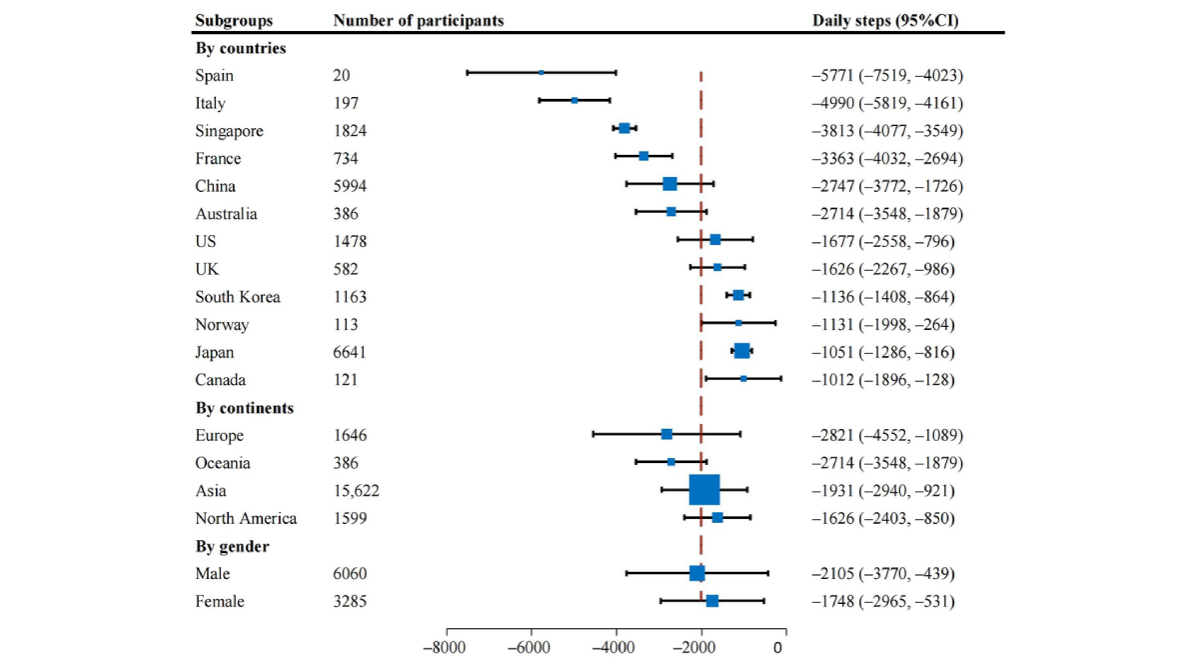
Pooled subgroup results of reduction in daily steps during the confinement period of the COVID-19 pandemic.

Country-specific analyses (Figure 5) showed that the most significant decline in daily steps occurred in subjects from Spain (MD=–5771, 95% CI –7519 to –4023), followed by Italy (MD=–4990, 95% CI –5819 to –4161) and Singapore (MD=–3813, 95% CI –4077 to –3549). The decline in daily steps was less in subjects from Canada (MD=–1012, 95% CI –1896 to –128), Japan (MD=–1051, 95% CI –1286 to –816), and Norway (MD=–1131, 95% CI –1998 to –264). In terms of continent, European subjects presented the most significant reduction in daily steps but with a wide CI (MD=–2821, 95% CI –4552 to –1089), followed by subjects from Oceania (MD=–2714, 95% CI –3548 to –1879) and Asia (MD=–1931, 95% CI –2940 to –921), while subjects from North America presented the smallest decline in daily steps (MD=–1626, 95% CI –2403 to –850). Of the studies that provided gender-specific data [20,41,43,45-47,52], no apparent difference was observed in the change in daily steps between men (MD=–2105, 95% CI –3770 to –439) and women (MD=–1748, 95% CI –2965 to –531).

## Discussion

### Principal Results

Our study quantified the change in daily steps during the confinement period of COVID-19. The proportion of studies with subjects with optimal daily steps (ie, ≥7000 steps/day) declined from 70% before the pandemic to 25% during the confinement period, and the average number of daily steps was reduced by 2000. Our results also suggest a possible inequality in physical activity across countries, whether before or during the pandemic. However, given that most included studies lacked sufficient nationwide representativeness, this difference should be interpreted cautiously. Furthermore, there was no apparent gender difference in the decline in daily steps before and during the confinement period of COVID-19.

### Possible Explanations and Clinical Implications

The population-level trends in daily steps during the confinement period of COVID-19 may reflect public panic about the virus and adherence to confinement measures (ie, physical distancing and sheltering in place) [55,56]. Our findings are not surprising given that COVID-19 restriction measures deprived the general population of many opportunities for physical activity, such as commuting, shopping, sports, and vacations or trips. In addition, the observed differences in the change in daily steps across studies may reflect regional variations in enforcement and behavioral changes [57,58]. Socioeconomic inequalities might also influence such variations among regions and disparities in the ability to engage in or access recreational physical activity within areas [59].

Daily steps are strongly associated with health outcomes [7-16]. Recent studies indicate that over 7000 steps per day is the critical physical activity standard for minimizing the risk of all-cause mortality [12,13]. Moreover, the risk of cardiovascular events increases by 8% for every 2000 steps per day decrement [14]. We found that daily steps declined by 2000, and half of the included studies reported that average daily steps declined to below 7000 during the confinement period of the COVID-19 pandemic. Such findings imply that the pandemic further exacerbated the ever-increasing prevalence of low-level physical activity [60,61], emphasizing the necessity of adopting appropriate measures to reverse this trend. Furthermore, this meta-analysis focused on observational studies based on monitoring devices. Since monitoring devices may encourage users to engage in physical activities [6], the number of daily steps during the confinement period might have been even lower in the population than was currently observed in the samples. In addition, the impact of COVID-19 on daily steps may persist in the long term. Although some researchers report that daily steps gradually recovered to the prepandemic level after the lifting of restriction measures [49,62], more studies argue that worldwide daily steps remain depressed compared with the prepandemic level (ie, the immediate 2 years before the outbreak of COVID-19) [59,63-66]. Thus, the consequences of long-term physical inactivity, which may be further exacerbated, should be addressed.

### Comparison With Previous Work

Our finding of a reduction in daily steps during the confinement period of the pandemic is consistent with findings from previous studies using different physical activity metrics [67,68]. An earlier meta-analysis found that sedentary time significantly increased, by 126.9 minutes per day in adults and by 46.9 minutes per day in the elderly, during the confinement period of the pandemic [67]. Another systematic review reported that most of the included studies revealed a decrease in physical activity in most participants [68]. All studies showed an increased sedentary time in healthy adults and those with medical conditions during the lockdown period [68]. According to this evidence, public health messages about staying active during the pandemic appear to have had little effect on the population’s engagement in physical activity [69]. In addition, 2 studies explored gender differences in the change in physical activity during the COVID-19 pandemic but had inconsistent conclusions [24,25]. One study found that men had more clearly decreased moderate-vigorous physical activity and increased sedentary behaviors than women during the pandemic [24]. However, another study failed to show significant differences between men and women in self-reported decline in physical activity [25]. Our meta-analysis adopted a primary indicator of physical activity, ie, daily steps, and found that daily steps declined substantially during the COVID-19 confinement period. Such a decline varied by region but not by gender. These results are consistent with previous studies [24,25,67,68].

Based on data collected from 187 countries and districts by a smartphone app, Tison and colleagues [59] found that mean daily steps declined by 5.5% (287 steps) within 10 days of the pandemic declaration by the World Health Organization and by 27.3% (1432 steps) within 30 days. The authors also reported noticeable differences across regions; for example, Italy, France, Brazil, and Iran exhibited a more than 40% maximum decrease, while Sweden exhibited a maximum reduction of daily steps of less than 10% [59]. Furthermore, regional differences were significant. For example, several studies showed that the decline in daily steps during confinement varied considerably among Chinese adults from different provinces [40,41,43]. One nationwide study in Japan found that daily steps declined significantly after the declaration of a state of emergency in urban areas but not rural areas [62].

### Limitations

Several limitations should be acknowledged. First, daily step data are lacking from Africa and South America, limiting the generalizability of our findings. Second, when comparing regional differences across countries, we could not rule out potential sampling bias, because most of the included studies lacked sufficient nationwide representativeness. Therefore, future studies are needed to verify our findings. Third, since long-term data from the period that confinement was lifted are not suitable for inclusion in a meta-analysis, we could not directly assess the long-term impact of COVID-19 on physical activity levels using meta-analysis techniques. As more published data become available [59,63-66], this will be an important direction for future studies. Fourth, we did not examine the impact of reduced daily steps on health outcomes, owing to a lack of relevant data. Future studies are expected to address this crucial issue. Finally, there was considerable study heterogeneity. Potential explanations include discrepancies in confinement measures across countries or districts and in the adherence to policy of residents, as well as unclear or different time frames over which the average daily steps were calculated, the possibility that monitoring devices were changed in the different reporting periods, and different durations of observation.

### Conclusion

In summary, we found that daily steps declined substantially during the COVID-19 confinement period. Our findings indicate that COVID-19 further exacerbated the ever-increasing prevalence of low levels of physical activity, emphasizing the necessity of adopting appropriate measures to reverse this trend. Further research is required to monitor the consequence of long-term physical inactivity.

## Appendix

Multimedia Appendix 1 MOOSE checklist.

Multimedia Appendix 2 Supplementary methods and results.

## Abbreviations

ISRCTN: International Standard Randomized Controlled Trial Number Register
MD: mean difference
MOOSE: Meta-analysis Of Observational Studies in Epidemiology
NOS: Newcastle-Ottawa Scale
PRISMA: Preferred Reporting Items for Systematic Reviews and Meta-Analysis

## References

[1] Martinez-Gomez D, Esteban-Cornejo I, Lopez-Garcia E, García-Esquinas Esther, Sadarangani KP, Veiga OL, Rodriguez-Artalejo F (2020). Physical activity less than the recommended amount may prevent the onset of major biological risk factors for cardiovascular disease: a cohort study of 198 919 adults. Br J Sports Med.

[2] Iso-Markku P, Kujala UM, Knittle K, Polet J, Vuoksimaa E, Waller K (2022). Physical activity as a protective factor for dementia and Alzheimer's disease: systematic review, meta-analysis and quality assessment of cohort and case-control studies. Br J Sports Med.

[3] Tarp J, Fagerland MW, Dalene KE, Johannessen JS, Hansen BH, Jefferis BJ, Whincup PH, Diaz KM, Hooker S, Howard VJ, Chernofsky A, Larson MG, Spartano NL, Vasan RS, Dohrn I, Hagströmer Maria, Edwardson C, Yates T, Shiroma EJ, Dempsey PC, Wijndaele K, Anderssen SA, Lee I, Ekelund U (2022). Device-measured physical activity, adiposity and mortality: a harmonised meta-analysis of eight prospective cohort studies. Br J Sports Med.

[4] Zhao M, Veeranki SP, Li S, Steffen LM, Xi B (2019). Beneficial associations of low and large doses of leisure time physical activity with all-cause, cardiovascular disease and cancer mortality: a national cohort study of 88,140 US adults. Br J Sports Med.

[5] 2018 Physical Activity Guidelines Advisory Committee Scientific Report. Department of Health and Human Services.

[6] Bravata DM, Smith-Spangler C, Sundaram V, Gienger AL, Lin N, Lewis R, Stave CD, Olkin I, Sirard JR (2007). Using pedometers to increase physical activity and improve health: a systematic review. JAMA.

[7] Lin H, Sardana M, Zhang Y, Liu C, Trinquart L, Benjamin EJ, Manders ES, Fusco K, Kornej J, Hammond MM, Spartano NL, Pathiravasan CH, Kheterpal V, Nowak C, Borrelli B, Murabito JM, McManus DD (2020). Association of habitual physical activity with cardiovascular disease risk. Circ Res.

[8] Fretts Amanda M, Howard Barbara V, McKnight Barbara, Duncan Glen E, Beresford Shirley A A, Calhoun Darren, Kriska Andrea M, Storti Kristi L, Siscovick David S (2012). Modest levels of physical activity are associated with a lower incidence of diabetes in a population with a high rate of obesity: the strong heart family study. Diabetes Care.

[9] Dwyer T, Ponsonby A, Ukoumunne OC, Pezic A, Venn A, Dunstan D, Barr E, Blair S, Cochrane J, Zimmet P, Shaw J (2011). Association of change in daily step count over five years with insulin sensitivity and adiposity: population based cohort study. BMJ.

[10] Lee I, Shiroma EJ, Kamada M, Bassett DR, Matthews CE, Buring JE (2019). Association of step volume and intensity with all-cause mortality in older women. JAMA Intern Med.

[11] Saint-Maurice PF, Troiano RP, Bassett DR, Graubard BI, Carlson SA, Shiroma EJ, Fulton JE, Matthews CE (2020). Association of daily step count and step intensity with mortality among US adults. JAMA.

[12] Paluch AE, Bajpai S, Bassett DR, Carnethon MR, Ekelund U, Evenson KR, Galuska DA, Jefferis BJ, Kraus WE, Lee I, Matthews CE, Omura JD, Patel AV, Pieper CF, Rees-Punia E, Dallmeier D, Klenk J, Whincup PH, Dooley EE, Pettee Gabriel K, Palta P, Pompeii LA, Chernofsky A, Larson MG, Vasan RS, Spartano N, Ballin M, Nordström Peter, Nordström Anna, Anderssen SA, Hansen BH, Cochrane JA, Dwyer T, Wang J, Ferrucci L, Liu F, Schrack J, Urbanek J, Saint-Maurice PF, Yamamoto N, Yoshitake Y, Newton RL, Yang S, Shiroma EJ, Fulton JE, Steps for Health Collaborative (2022). Daily steps and all-cause mortality: a meta-analysis of 15 international cohorts. Lancet Public Health.

[13] Paluch AE, Gabriel KP, Fulton JE, Lewis CE, Schreiner PJ, Sternfeld B, Sidney S, Siddique J, Whitaker KM, Carnethon MR (2021). Steps per day and all-cause mortality in middle-aged adults in the coronary artery risk development in young adults study. JAMA Netw Open.

[14] Yates T, Haffner SM, Schulte PJ, Thomas L, Huffman KM, Bales CW, Califf RM, Holman RR, McMurray JJV, Bethel MA, Tuomilehto J, Davies MJ, Kraus WE (2014). Association between change in daily ambulatory activity and cardiovascular events in people with impaired glucose tolerance (NAVIGATOR trial): a cohort analysis. Lancet.

[15] Breen L, Stokes KA, Churchward-Venne TA, Moore DR, Baker SK, Smith K, Atherton PJ, Phillips SM (2013). Two weeks of reduced activity decreases leg lean mass and induces "anabolic resistance" of myofibrillar protein synthesis in healthy elderly. J Clin Endocrinol Metab.

[16] Bowden Davies KA, Norman JA, Thompson A, Mitchell KL, Harrold JA, Halford JCG, Wilding JPH, Kemp GJ, Cuthbertson DJ, Sprung VS (2021). Short-term physical inactivity induces endothelial dysfunction. Front Physiol.

[17] Coronavirus: the world in lockdown in maps and charts. British Broadcasting Corporation.

[18] Coronavirus disease (COVID-19) pandemic. World Health Organization.

[19] Wang Y, Zhang Y, Bennell K, White DK, Wei J, Wu Z, He H, Liu S, Luo X, Hu S, Zeng C, Lei G (2020). Physical distancing measures and walking activity in middle-aged and older residents in Changsha, China, during the COVID-19 epidemic period: longitudinal observational study. J Med Internet Res.

[20] Ong Ju Lynn, Lau TeYang, Massar Stijn A A, Chong Zhi Ting, Ng Ben K L, Koek Daphne, Zhao Wanting, Yeo B T Thomas, Cheong Karen, Chee Michael W L (2021). COVID-19-related mobility reduction: heterogenous effects on sleep and physical activity rhythms. Sleep.

[21] Giuntella O, Hyde K, Saccardo S, Sadoff S (2021). Lifestyle and mental health disruptions during COVID-19. Proc Natl Acad Sci U S A.

[22] To QG, Duncan MJ, Van Itallie A, Vandelanotte C (2021). Impact of COVID-19 on physical activity among 10,000 steps members and engagement with the program in Australia: prospective study. J Med Internet Res.

[23] Pépin Jean Louis, Bruno RM, Yang R, Vercamer V, Jouhaud P, Escourrou P, Boutouyrie P (2020). Wearable activity trackers for monitoring adherence to home confinement during the COVID-19 pandemic worldwide: data aggregation and analysis. J Med Internet Res.

[24] Castañeda-Babarro Arkaitz, Arbillaga-Etxarri A, Gutiérrez-Santamaría Borja, Coca A (2020). Physical activity change during COVID-19 confinement. Int J Environ Res Public Health.

[25] Wilke J, Mohr L, Tenforde AS, Edouard P, Fossati C, González-Gross Marcela, Sánchez Ramírez Celso, Laiño Fernando, Tan B, Pillay JD, Pigozzi F, Jimenez-Pavon D, Novak B, Jaunig J, Zhang M, van Poppel M, Heidt C, Willwacher S, Yuki G, Lieberman DE, Vogt L, Verhagen E, Hespanhol L, Hollander K (2021). A Pandemic within the pandemic? physical activity levels substantially decreased in countries affected by COVID-19. Int J Environ Res Public Health.

[26] Stroup DF, Berlin J A, Morton S C, Olkin I, Williamson G D, Rennie D, Moher D, Becker B J, Sipe T A, Thacker S B (2000). Meta-analysis Of Observational Studies in Epidemiology: a proposal for reporting. Meta-analysis Of Observational Studies in Epidemiology (MOOSE) group. JAMA.

[27] Page MJ, Moher D, Bossuyt PM, Boutron I, Hoffmann TC, Mulrow CD, Shamseer L, Tetzlaff JM, Akl EA, Brennan SE, Chou R, Glanville J, Grimshaw JM, Hróbjartsson Asbjørn, Lalu MM, Li T, Loder EW, Mayo-Wilson E, McDonald S, McGuinness LA, Stewart LA, Thomas J, Tricco AC, Welch VA, Whiting P, McKenzie JE (2021). PRISMA 2020 explanation and elaboration: updated guidance and exemplars for reporting systematic reviews. BMJ.

[28] Wells GA, Shea B, O'Connell D, Peterson J, Welch V, Losos M, Tugwell P (2009). The Newcastle-Ottawa Scale (NOS) for assessing the quality of nonrandomised studies in meta-analyses. Ottawa Hospital Research Institute.

[29] Ceban F, Nogo D, Carvalho IP, Lee Y, Nasri F, Xiong J, Lui LMW, Subramaniapillai M, Gill H, Liu RN, Joseph P, Teopiz KM, Cao B, Mansur RB, Lin K, Rosenblat JD, Ho RC, McIntyre RS (2021). Association between mood disorders and risk of COVID-19 infection, hospitalization, and death: a systematic review and meta-analysis. JAMA Psychiatry.

[30] Smith KN, Baynard T, Fischbach PS, Hankins JS, Hsu LL, Murphy PM, Ness KK, Radom-Aizik S, Tang A, Liem RI (2022). Safety of maximal cardiopulmonary exercise testing in individuals with sickle cell disease: a systematic review. Br J Sports Med.

[31] Li Ling, Shen Jiantong, Bala Malgorzata M, Busse Jason W, Ebrahim Shanil, Vandvik Per Olav, Rios Lorena P, Malaga German, Wong Evelyn, Sohani Zahra, Guyatt Gordon H, Sun Xin (2014). Incretin treatment and risk of pancreatitis in patients with type 2 diabetes mellitus: systematic review and meta-analysis of randomised and non-randomised studies. BMJ.

[32] Deeks J, Higgins J, Altman D, Higgins J, Green S (2011). Chapter 9: Analysing data and undertaking meta-analyses. Cochrane Handbook for Systematic Reviews of Interventions. Version 5.

[33] GetData Graph Digitizer.

[34] Wojtyniak J, Britz H, Selzer D, Schwab M, Lehr T (2020). Data digitizing: accurate and precise data extraction for quantitative systems pharmacology and physiologically-based pharmacokinetic modeling. CPT Pharmacometrics Syst Pharmacol.

[35] Higgins JPT, Thompson SG (2002). Quantifying heterogeneity in a meta-analysis. Stat Med.

[36] Egger M, Davey Smith G, Schneider M, Minder C (1997). Bias in meta-analysis detected by a simple, graphical test. BMJ.

[37] Dunton GF, Wang SD, Do B, Courtney J (2020). Early effects of the COVID-19 pandemic on physical activity locations and behaviors in adults living in the United States. Prev Med Rep.

[38] Karageorghis CI, Bird JM, Hutchinson JC, Hamer M, Delevoye-Turrell YN, Guérin Ségolène M R, Mullin EM, Mellano KT, Parsons-Smith RL, Terry VR, Terry PC (2021). Physical activity and mental well-being under COVID-19 lockdown: a cross-sectional multination study. BMC Public Health.

[39] Woodruff SJ, Coyne P, St-Pierre Emily (2021). Stress, physical activity, and screen-related sedentary behaviour within the first month of the COVID-19 pandemic. Appl Psychol Health Well Being.

[40] Ding D, Cheng M, Del Pozo Cruz Borja, Lin T, Sun S, Zhang L, Yang Q, Ma Z, Wang J, Jia Y, Shi Y (2021). How COVID-19 lockdown and reopening affected daily steps: evidence based on 164,630 person-days of prospectively collected data from Shanghai, China. Int J Behav Nutr Phys Act.

[41] He M, Xian Y, Lv X, He J, Ren Y (2021). Changes in body weight, physical activity, and lifestyle during the semi-lockdown period after the outbreak of COVID-19 in China: an online survey. Disaster Med Public Health Prep.

[42] Ji Y, Shi Y, Zhou J, Li X, Qin R, Zhu Q (2022). Analysis on the change of college students' life pattern and its impact during the COVID-19 outbreak in China. Am J Health Behav.

[43] Zhu Y, Wang Z, Maruyama H, Onoda K, Huang Q, Hu C, Zhou Y (2021). Effect of the COVID-19 lockdown period on the physical condition, living habits, and physical activity of citizens in Beijing, China. J Phys Ther Sci.

[44] Buoite Stella Alex, AjČeviĆ Miloš, Furlanis Giovanni, Cillotto Tommaso, Menichelli Alina, Accardo Agostino, Manganotti Paolo (2021). Smart technology for physical activity and health assessment during COVID-19 lockdown. J Sports Med Phys Fitness.

[45] Azuma K, Nojiri T, Kawashima M, Hanai A, Ayaki M, Tsubota K, TRF-Japan Study Group (2021). Possible favorable lifestyle changes owing to the coronavirus disease 2019 (COVID-19) pandemic among middle-aged Japanese women: An ancillary survey of the TRF-Japan study using the original "Taberhythm" smartphone app. PLoS One.

[46] Obuchi SP, Kawai H, Ejiri M, Ito K, Murakawa K (2021). Change in outdoor walking behavior during the coronavirus disease pandemic in Japan: A longitudinal study. Gait Posture.

[47] Sato K, Sakata R, Murayama C, Yamaguchi M, Matsuoka Y, Kondo N (2021). Changes in work and life patterns associated with depressive symptoms during the COVID-19 pandemic: an observational study of health app (CALO mama) users. Occup Environ Med.

[48] Yamada Y, Namba H, Date H, Kitayama S, Nakayama Y, Kimura M, Fujita H, Miyachi M (2023). Regional difference in the impact of COVID-19 pandemic on domain-specific physical activity, sedentary behavior, sleeping time, and step count: web-based cross-sectional nationwide survey and accelerometer-based observational study. JMIR Public Health Surveill.

[49] Henriksen A, Johannessen E, Hartvigsen G, Grimsgaard S, Hopstock LA (2021). Consumer-based activity trackers as a tool for physical activity monitoring in epidemiological studies during the COVID-19 pandemic: development and usability study. JMIR Public Health Surveill.

[50] Park J, Yoo E, Kim Y, Lee J (2021). What happened pre- and during COVID-19 in South Korea? Comparing physical activity, sleep time, and body weight status. Int J Environ Res Public Health.

[51] Sañudo B, Fennell C, Sánchez-Oliver AJ (2020). Objectively-assessed physical activity, sedentary behavior, smartphone use, and sleep patterns pre- and during-COVID-19 quarantine in young adults from Spain. Sustainability.

[52] Bird JM, Karageorghis CI, Hamer M (2021). Relationships among behavioural regulations, physical activity, and mental health pre- and during COVID-19 UK lockdown. Psychol Sport Exerc.

[53] Hudgins BL, Kurti SP, Edwards ES, Hargens TA (2021). The impact of the COVID-19 pandemic on physical activity habits at a residential university. J Am Coll Health.

[54] Gollwitzer A, McLoughlin K, Martel C, Marshall J, Höhs JM, Bargh JA (2021). Linking self-reported social distancing to real-world behavior during the COVID-19 pandemic. Soc Psychol Personal Sci.

[55] Luo F, Ghanei Gheshlagh R, Dalvand S, Saedmoucheshi S, Li Q (2021). Systematic review and meta-analysis of fear of COVID-19. Front Psychol.

[56] Bargain O, Aminjonov U (2020). Trust and compliance to public health policies in times of COVID-19. J Public Econ.

[57] Pak A, McBryde E, Adegboye OA (2021). Does high public trust amplify compliance with stringent COVID-19 government health guidelines? A multi-country analysis using data from 102,627 individuals. Risk Manag Healthc Policy.

[58] Fridman I, Lucas N, Henke D, Zigler CK (2020). Association between public knowledge about COVID-19, trust in information sources, and adherence to social distancing: cross-sectional survey. JMIR Public Health Surveill.

[59] Tison GH, Avram R, Kuhar P, Abreau S, Marcus GM, Pletcher MJ, Olgin JE (2020). Worldwide effect of COVID-19 on physical activity: a descriptive study. Ann Intern Med.

[60] Physical activity. World Health Organization.

[61] Hall G, Laddu DR, Phillips SA, Lavie CJ, Arena R (2021). A tale of two pandemics: How will COVID-19 and global trends in physical inactivity and sedentary behavior affect one another?. Prog Cardiovasc Dis.

[62] Yamada Y, Yoshida T, Nakagata T, Nanri H, Miyachi M (2021). Letter to the editor: Age, sex, and regional differences in the effect of COVID-19 pandemic on objective physical activity in Japan: A 2-year nationwide longitudinal study. J Nutr Health Aging.

[63] Tison GH, Barrios J, Avram R, Kuhar P, Bostjancic B, Marcus GM, Pletcher MJ, Olgin JE (2022). Worldwide physical activity trends since COVID-19 onset. Lancet Glob Health.

[64] Lawhun Costello V, Chevance G, Wing D, Mansour-Assi SJ, Sharp S, Golaszewski NM, Young EA, Higgins M, Ibarra A, Larsen B, Godino JG (2021). Impact of the COVID-19 pandemic on objectively measured physical activity and sedentary behavior among overweight young adults: yearlong longitudinal analysis. JMIR Public Health Surveill.

[65] Cornelius T, Denes A, Webber KT, Guest C, Goldsmith J, Schwartz JE, Gorin AA (2022). Relationship quality and objectively measured physical activity before and after implementation of COVID-19 stay-home orders. J Health Psychol.

[66] Barbieri PN, Giuntella O, Saccardo S, Sadoff S (2021). Lifestyle and mental health 1 year into COVID-19. Sci Rep.

[67] Runacres A, Mackintosh KA, Knight RL, Sheeran L, Thatcher R, Shelley J, McNarry MA (2021). Impact of the COVID-19 pandemic on sedentary time and behaviour in children and adults: a systematic review and meta-analysis. Int J Environ Res Public Health.

[68] Stockwell S, Trott M, Tully M, Shin J, Barnett Y, Butler L, McDermott D, Schuch F, Smith L (2021). Changes in physical activity and sedentary behaviours from before to during the COVID-19 pandemic lockdown: a systematic review. BMJ Open Sport Exerc Med.

[69] Staying physically active during the COVID-19 pandemic. American College of Sports Medicine.

