# Supplementary material for: The COVID-19 Pandemic and Daily Steps in the General Population: Meta-analysis of Observational Studies
Source: JMIR Public Health Surveill. 2023 May 30;9:e40650. doi: 10.2196/40650 (PMC10231625; doi:10.2196/40650)
Supplement: Multimedia Appendix 2 [file publichealth_v9i1e40650_app2.docx]

**Web Appendix 1**

**JMIR Public Health and Surveillance**

**The COVID-19 Pandemic and Daily Steps in the General Population: A Meta-analysis of Observational Studies**

**Table of Contents**

[**Figure S1** Funnel plot of the change in daily steps during the confinement period of the COVID-19 pandemic **1**](#_Toc128136244)

[**Figure S2** Egger’s publication bias plot of the change in daily steps during the confinement period of the COVID-19 pandemic **2**](#_Toc128136245)

[**Figure S3** Sensitivity analysis by omitted study one by one **3**](#_Toc128136246)

[**Table S1** Quality assessment for studies included in meta-analysis **4**](#_Toc128136247)

[**Search Strategy** **5**](#_Toc128136248)

[**The modified Newcastle-Ottawa Scale (NOS)** **6**](#_Toc128136249)


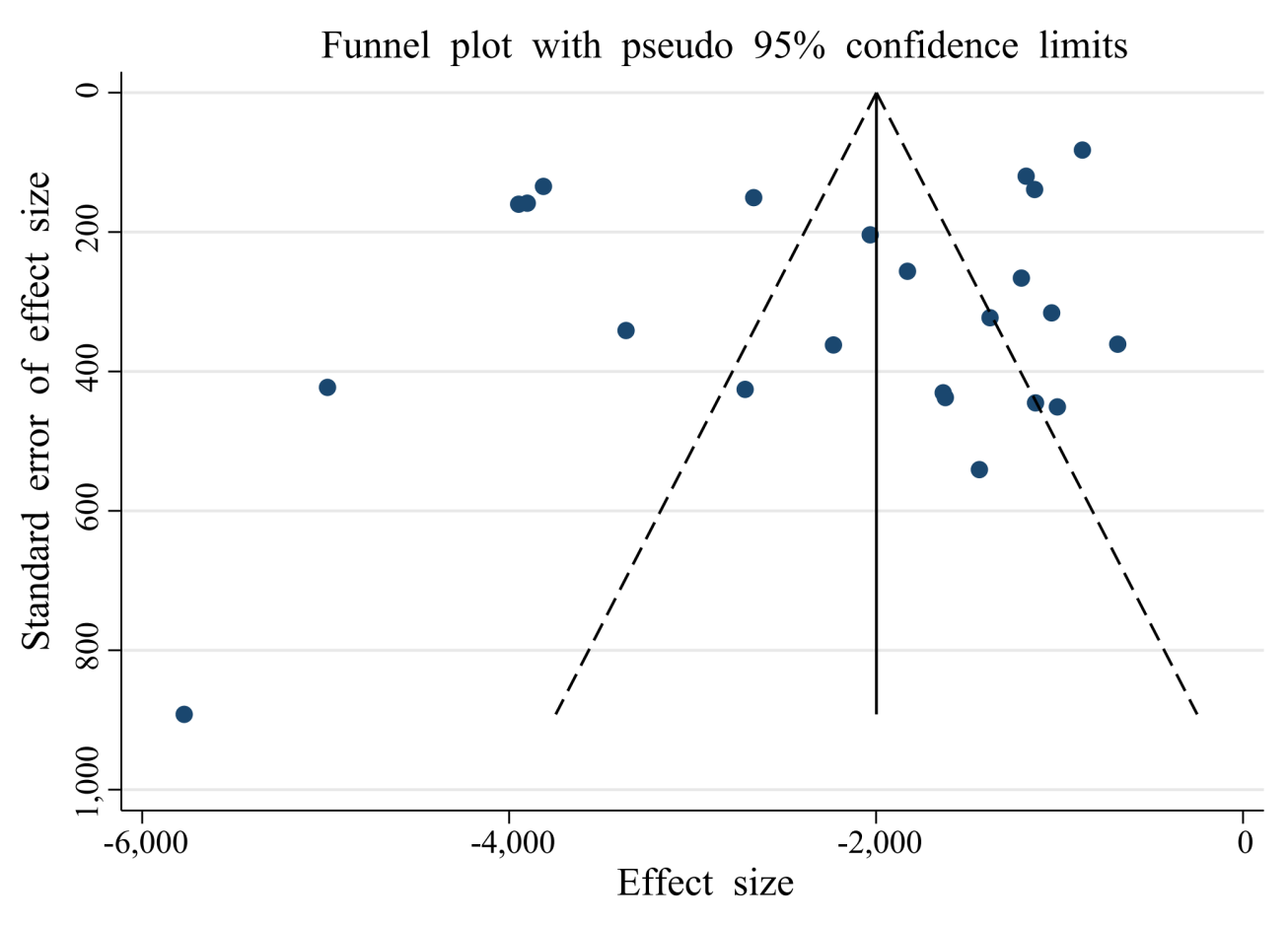


# Figure S1 Funnel plot of the change in daily steps during the confinement period of the COVID-19 pandemic

One study provided 4 separate data from different countries, so a total of 23 data points were shown.


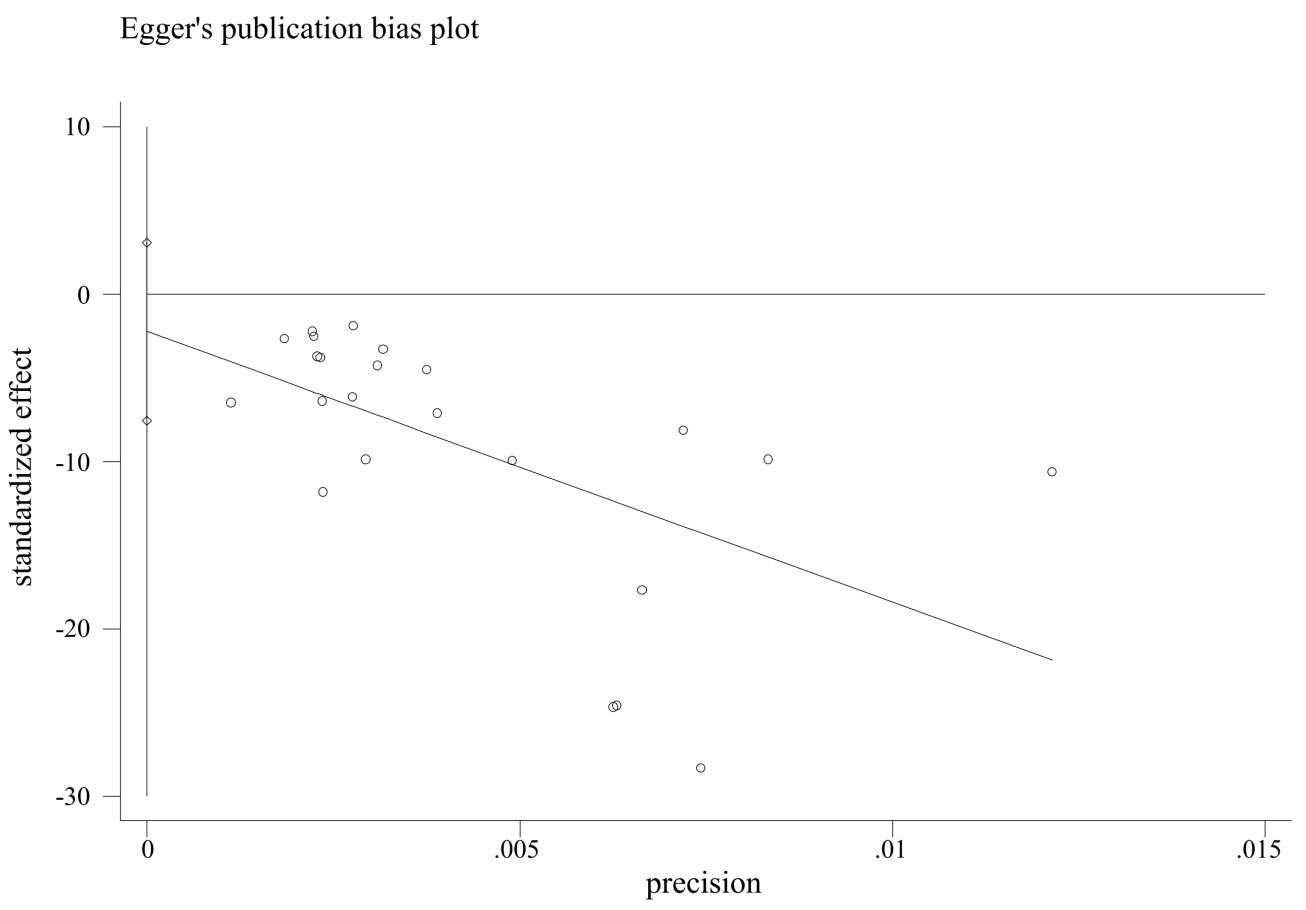


# Figure S2 Egger’s publication bias plot of the change in daily steps during the confinement period of the COVID-19 pandemic

One study provided 4 separate data from different countries, so a total of 23 data points were show.


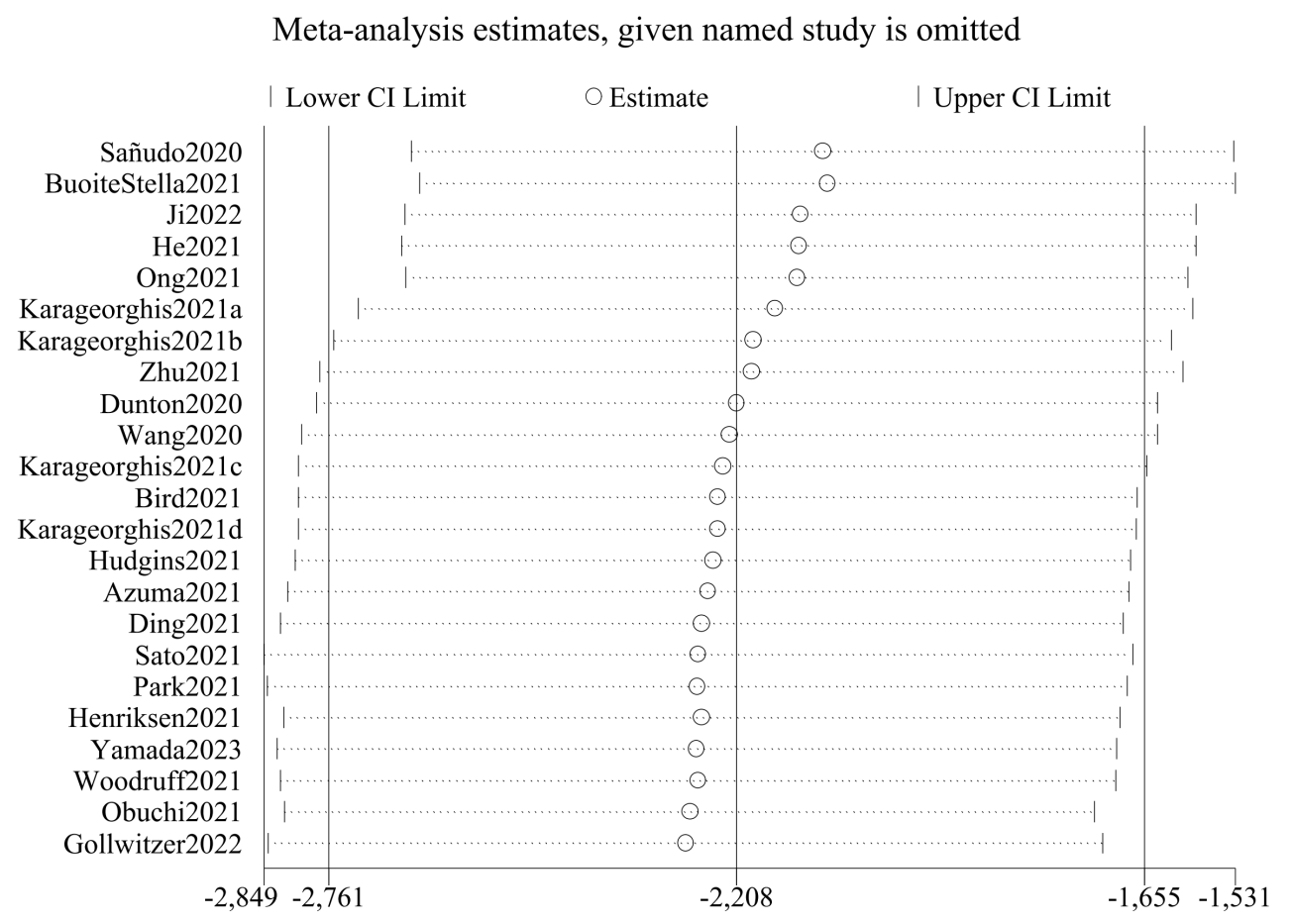


# Figure S3 Sensitivity analysis by omitted study one by one

CI, confidence interval.

# Table S1 Quality assessment for studies included for meta-analysis

| **Study** | **Selection** | | | **Comparability** | **Outcome** | | **Total “star”**  **（Max 7）** | **Methodological quality** |
| --- | --- | --- | --- | --- | --- | --- | --- | --- |
|  | Representativeness of exposed cohort | Selection of non-exposed cohort | Ascertainment of the exposure | Comparability | Assessment of the outcome | Statistics |  |  |
| Azuma 2021 | 0 | 1 | 1 | 2 | 1 | 1 | 6 | high |
| Bird 2021 | 0 | 1 | 1 | 1 | 0 | 0 | 3 | low |
| Buoite Stella 2021 | 0 | 1 | 1 | 2 | 0 | 1 | 5 | moderate |
| Ding 2021 | 0 | 1 | 1 | 2 | 1 | 1 | 6 | high |
| Dunton 2020 | 0 | 1 | 1 | 1 | 1 | 1 | 5 | moderate |
| Gollwitzer 2022 | 0 | 1 | 0 | 2 | 1 | 1 | 5 | moderate |
| He 2020 | 0 | 1 | 1 | 1 | 0 | 1 | 4 | low |
| Henriksen 2021 | 0 | 1 | 1 | 1 | 1 | 1 | 5 | moderate |
| Hudgins 2021 | 0 | 1 | 1 | 1 | 1 | 1 | 5 | moderate |
| Ji 2022 | 0 | 1 | 0 | 1 | 0 | 1 | 3 | low |
| Karageorghis 2021 | 0 | 1 | 1 | 1 | 0 | 1 | 4 | low |
| Obuchi 2021 | 1 | 1 | 1 | 2 | 1 | 1 | 7 | high |
| Ong 2021 | 0 | 1 | 1 | 1 | 1 | 1 | 5 | moderate |
| Park 2021 | 0 | 1 | 1 | 1 | 0 | 1 | 4 | low |
| Sañudo 2020 | 0 | 1 | 1 | 1 | 1 | 1 | 5 | moderate |
| Sato 2021 | 0 | 1 | 1 | 1 | 1 | 1 | 5 | moderate |
| Wang 2020 | 0 | 1 | 1 | 2 | 1 | 1 | 6 | high |
| Woodruff 2021 | 0 | 1 | 1 | 1 | 0 | 1 | 4 | low |
| Yamada 2023 | 1 | 1 | 1 | 0 | 1 | 1 | 5 | moderate |
| Zhu 2021 | 0 | 1 | 0 | 1 | 0 | 1 | 3 | low |

# Search Strategy

**PubMed**

#1 (COVID-19 OR “Novel Coronavirus” OR 2019-nCoV OR SARS-CoV-2 OR “coronavirus disease 2019”)[Title/Abstract]

#2 (accelerometer* OR pedometer* OR “step per day” OR “steps per day” OR “daily step” OR “daily steps” OR steps?day OR step?day OR “step count?day” OR counts?day OR “steps per day” OR “step counts” OR walk OR walking OR ambulation OR “physical activity”)[Title/Abstract]

#3 #1 and #2

**Embase**

#1 “coronavirus disease 2019”:ti,ab,kw OR “severe acute respiratory syndrome coronavirus 2”:ti,ab,kw

#2 “physical activity”:ti,ab,kw OR accelerometer:ti,ab,kw OR pedometer:ti,ab,kw OR “steps per day”:ti,ab,kw OR “daily steps”:ti,ab,kw OR “step per day”:ti,ab,kw OR “steps count”:ti,ab,kw OR “step counts”:ti,ab,kw OR “counts/d”:ti,ab,kw OR walking:ti,ab,kw OR mobilization:ti,ab,kw

#3 #1 and #2

**Web of Science**

#1 COVID-19 OR “Novel Coronavirus” OR 2019-nCoV OR SARS-CoV-2 OR “coronavirus disease 2019”(Abstract)

#2 accelerometer* OR pedometer* OR “step per day” OR “steps per day” OR “daily step” OR “daily steps” OR steps?day OR step?day OR “step count?day” OR counts?day OR “steps per day” OR “step counts” OR walk OR walking OR ambulation OR “physical activity”(Abstract)

#3 #1 and #2

# The modified Newcastle-Ottawa Scale (NOS)

Note: A study can be awarded a maximum of one star (★) for each numbered item within the Selection and Outcome categories. A maximum of two stars can be given for Comparability

Maximum: 7 stars

Methodological Quality Rating: high = 6-7 stars, moderate = 5 stars, low = 4 or fewer stars

| **A. Selection** (Maximum 3 stars) | |
| --- | --- |
| 1. Representativeness of the exposed cohort | 1. Truly representative of the average adults in the community (e.g., nationwide database) ★ 2. Somewhat representative of the average adults in the community (e.g., city-wide) ★ 3. Selected group of users (e.g., students, volunteers, wearable device users) 4. No description of the derivation of the cohort |
| 1. Selection of the non-exposed cohort | 1. Drawn from the same community/database as the exposed cohort ★ 2. Drawn from a different source 3. No description of the derivation of the non-exposed cohort |
| 1. Ascertainment of exposure | 1. Official policy for controlling spread of pandemic (e.g., keep physical distance, suspend the collective activities, lockdown) ★ 2. No description |
| **B. Comparability** (Maximum 2 stars) | |
| 1. Comparability of cohorts based on the design or analysis | The subjects in different outcome groups are comparable, based on the study design or analysis. Confounding factors are controlled.   1. Study controls for both age and sex ★ 2. Study controls for any additional factor ★ |
| **C. Outcome** (Maximum 2 stars) | |
| 1. Assessment of outcome | 1. Validated method to measure step count (e.g., wearable devices or smartphone-based automatic data collection, with or without interactive interface) ★ 2. Self-report (e.g., smartphone-based self-report) 3. No description |
| 1. Statistics | 1. The number of daily step count was reported, and appropriate descriptive analysis was applied for the outcome ★ 2. Incomplete or no description |

**References:**

1. GA Wells, B Shea, D O'Connell, et al. The Newcastle-Ottawa Scale (NOS) for assessing the quality of nonrandomised studies in meta-analyses. Ottawa, ON: Ottawa Hospital Research Institute, 2009. <http://www.ohri.ca/programs/clinical_epidemiology/oxford.asp>. Accessed 11 November 2021.
2. Ceban F, Nogo D, Carvalho IP, et al. Association Between Mood Disorders and Risk of COVID-19 Infection, Hospitalization, and Death: A Systematic Review and Meta-analysis. *JAMA Psychiatry*. 2021;78(10):1079-1091. [10.1001/jamapsychiatry.2021.1818]
3. Li L, Shen J, Bala MM, et al. Incretin treatment and risk of pancreatitis in patients with type 2 diabetes mellitus: systematic review and meta-analysis of randomised and non-randomised studies. *BMJ*. 2014;348:g2366. [10.1136/bmj.g2366]
4. Smith KN, Baynard T, Fischbach PS, et al. Safety of maximal cardiopulmonary exercise testing in individuals with sickle cell disease: a systematic review. *Br J Sports Med*. 2021:bjsports-2021-104450. [10.1136/bjsports-2021-104450]
